# Mesoporous magnetic biochar derived from common reed (*Phragmites australis*) for rapid and efficient removal of methylene blue from aqueous media

**DOI:** 10.1007/s11356-024-33860-3

**Published:** 2024-06-13

**Authors:** Wael Ibrahim Mortada, Mahmoud Mohsen Ghaith, Nada Elsayed Khedr, Mostafa Ibrahim Ellethy, Alaa Waleed Mohsen, Amira Labib Shafik

**Affiliations:** 1 Urology and Nephrology Center, Mansoura University, Mansoura, Egypt; 2 Petrochemical Program, Chemistry Department, Faculty of Science, Mansoura University, Mansoura, Egypt

**Keywords:** Common reed, Magnetic biochar, Removal efficiency, Methylene blue

## Abstract

A novel mesoporous magnetic biochar (MBC) was prepared, using a randomly growing plant, i.e., common reed, as an exporter of carbon, and applied for removal of methylene blue (MB) from aqueous solutions. The prepared sorbent was characterized by nitrogen adsorption/desorption isotherm, saturation magnetization, pH of point of zero charges (pH_PZC_), Fourier-transform infrared spectroscopy (FT-IR), and scanning electron microscopy (SEM). The obtained MBC has a specific surface area of 94.2 m^2^ g^−1^ and a pore radius of 4.1 nm, a pore volume of 0.252 cm^3^ g^−1^, a saturation magnetization of 0.786 emu g^−1^, and a pH_PZC_ of 6.2. Batch adsorption experiments were used to study the impact of the physicochemical factors involved in the adsorption process. The findings revealed that MB removal by MBC was achieved optimally at pH 8.0, sorbent dosage of 1.0 g L^−1^, and contact time of 30 min. At these conditions, the maximum adsorption was 353.4 mg g^−1^. Furthermore, the adsorption isotherm indicated that the Langmuir pattern matched well with the experimental data, compared to the Freindlich model. The ∆*G* was − 6.7, − 7.1, and − 7.5 kJ mol^−1^, at 298, 308, and 318 K, respectively, indicating a spontaneous process. The values of ∆*H* and ∆*S* were 5.71 kJ mol^−1^ and 41.6 J mol^−1^ K^−1^, respectively, suggesting endothermic and the interaction between MB and MBC is van der Waals type. The absorbent was regenerated and reused for four cycles after elution with 0.1 mol L^−1^ of HCl. This study concluded that the magnetic biochar generated from common reed has tremendous promise in the practical use of removing MB from wastewater.

**Graphic Abstract:**

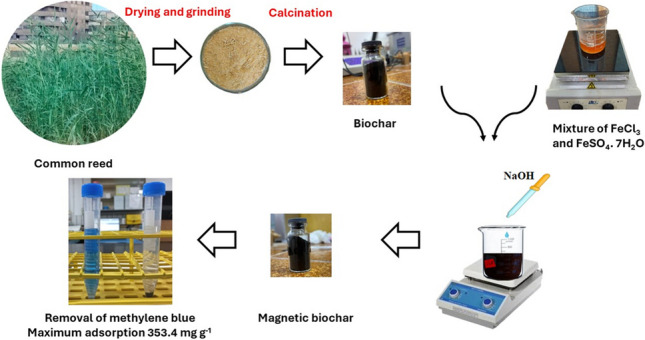

## Introduction

Cationic dyes, such as methylene blue (MB), are commonly utilized in numerous industries and their emission negatively impacts the purity of water, even in small amounts, interfering with light dispersion and affecting aquatic plant photosynthesis. They represent a considerable risk to aquatic life and humans because to their toxicity along with their poor biodegradability (Khan et al. 2022; Rehan et al. 2024).

Removal of MB has been accomplished using a variety of physicochemical processes, including adsorption (Abdelhameed et al. 2022; Mortada et al. 2023), membrane filtering (Li et al. 2017), co-precipitation (Guesmi et al. 2018), coagulation (Ihaddaden et al. 2022), and ion exchange (El-Moselhy and Kamal 2018). Even so, most of these technologies are impractical for dye removal due to their bio-refractory nature (Castro et al. 2018), high operational costs, or formation of more toxic biodegradation products (Mu et al. 2022). As a result, there is a need to find and develop inventive, economic, and environmentally friendly techniques for achieving a more effective method to remove dye from wastewater.

Adsorption, in particular, offers several advantages owing to its high efficacy, ease of use, and lack of toxic byproducts (Emam et al. 2023; Kadhom et al. 2020). Adsorbents are the primary component for adsorption, and a variety of resources have been employed for this reason. Clays were widely used owing to their availability as low-cost natural materials (Bello et al. 2015). However, identifying novel substances or changing existing ones was necessary to achieve effective adsorption capacity. For instance, activated carbon is widely documented in the field of dye and organic adsorption due to its outstanding efficacy, despite its relatively expensive cost, which limits its application (Reza et al. 2020). As a consequence, the need for less expensive but superior adsorbents as substitutes to activated carbon has increased; however, many of these adsorbents remain far from activated carbon regarding their adsorption efficiency (Alshandoudi et al. 2023; Sewu et al. 2017).

Recently, biomass agriculture waste has garnered significant interest for production of biochar (BC). These wastes are gets produced in large quantities as a result of the expanding food manufacturing sector and the increasing worldwide population (Karić et al. 2022). Biochar obtained from bamboo (Yang et al. 2014), rice husk (Saravanan et al. 2023), coconut coir (Le et al. 2021), cabbage waste (Sewu et al. 2017), pine wood (Rubio-Clemente et al. 2021), and litchi peel (Wu et al. 2020) were employed for removal of different organic dye from wastewater. In most of these studies, phase separation is performed by filtration or centrifugation steps, leading to extra time and costs. However, these approaches may fail to quantitatively separate the sorbent from the sample, resulting in secondary turbidity. To address the issues highlighted, magnetized activated carbon is proposed as a feasible alternative (Badi et al. 2018). Modification of activated carbon by Fe_3_O_4_ improves the chemical stability and recyclability of the sorbent used to remove various pollutants from wastewater (Azari et al. 2015).

The coprecipitation method is a simple and easy procedure for preparing MNPs. It offers benefits such as good yield, high product purity, no need for organic solvents, and low cost. This method is being utilized today to produce MNPs, particularly iron oxide. This approach involves reducing a mixture of Fe^2+^ and Fe^3+^ in alkaline medium at temperatures below 100 °C (Nawaz et al. 2019).

$${{Fe}^{2+}}_{(aq)} + {{Fe}^{3+}}_{(aq)} + {{8OH}^{-}}_{(aq)} \to {Fe}_{3}{O}_{4(s)} + {{4H}_{2}O}_{(1)}$$

Many investigators used the coprecipitation method for the modification of activated carbon by MNPs for water treatment purposes. Yang et al. prepared Fe_3_O_4_/carbon nanotube composite by co-precipitation method for removal of Cu^2+^ from aqueous media (Yang et al. 2018). In a recent study, commercial active carbon was fabricated by Fe_3_O_4_ for removal of Cr^6+^ and Mordant Violet 40 (Mohamed et al. 2024). Fe_3_O_4_/vine shoot-derived activated carbon nanocomposite was employed to remove Cr^6+^ from effluent (Mohamed et al. 2024).

Common reed (*Phragmites australis*) is a prevalent wetland plant and classified as the most widespread angiosperm (Mehner 2009). It is an intensely opportunistic plant that grows quickly and spreads widely. Common reed shifts native plants, diminishes biodiversity, provides very little benefit to ecosystems, and restricts waterways. On the other hand, the plant has many benefits, such as habitats for birds and invertebrates; mat; fodder; and building materials production, source of renewable energy, and treatment of sewage water (Andersen et al. 2021; Čížková et al. 2023; Köbbing et al. 2013).

In view of the preceding reasons, the goal of this work was to produce an effective adsorbent from a cheap, widely available, and accessible biomass material in many countries, i.e., common reed *(P. australis*). For this aim, in the first step of this investigation, the biochar was prepared from common reed by calcination. In the subsequent stage, the magnetic characteristics of biochar were acquired by formation of biochar iron oxide composite (MBC). The physicochemical and morphological properties of the prepared materials were investigated using X-ray diffraction (XRD), scanning electron microscopy/energy-dispersive X-ray analysis (SEM/EDS), Fourier-transform infrared spectra (FT-IR), and Brunauer–Emmett–Teller analyses (BET). Meanwhile, the adsorption behavior of MB onto MBC was systematically studied, and the adsorption mechanism was proposed based on the findings of pH_PZC_, FT-IR, and thermodynamic studies.

## Materials and methods

### Materials

Common reed samples were collected from Al-Khiarya village near Mansoura city. The sample was cleaned and rinsed with distillated water, cut into small pieces, dried at 90 °C, crushed, homogenized in a blender, sieved with a 200 mesh, and stored in a desiccator until used. Methylene blue (C_16_H_18_ClN_3_S), potassium hydroxide (KOH), hydrochloric acid (HCl), ferric chloride (FeCl_3_), and ferrous sulphate (FeSO_4_.7H_2_O) were purchased from Alpha Chemica (India).

### Preparation of magnetic biochar

Common reed BC was produced by calcination under N_2_ atmosphere at 400 °C with a heating rate of 5 °C/min for 120 min and then heating to 750 °C for 90 min (Shoaib et al. 2020). MBC was prepared according to Anyika et al. (Anyika et al. 2017) with minor modifications. Five grams of the prepared BC were suspended in a 50.0-mL distilled water. Freshly prepared solutions of FeCl_3_ (1.8 g in 130 mL) and FeSO_4_.7H_2_O (2.0 g in 15 mL) were mixed in a 250-mL flask and heated at about 65 °C followed by vigorous agitating with a magnetic stirrer. The produced suspension was added to the aqueous suspension of BC and stirred quietly for 20 min at the ambient temperature to ensure adequate mixing. After that, 10 mol L^−1^ of NaOH solution was added dropwise until obtaining pH ~ 11.0. The suspension was aged for 24 h, and the obtained precipitate was repeatedly rinsed with distilled water and ethanol until the pH of the filtrate reached ~ 7.0. The produced MBC was vacuum filtered and dried at 50 °C.

### Characterization

#### X-ray diffraction

The XRD patterns were obtained using Brucker Axs-D8 Advance diffractometer with CuKα monochromatic radiation. The samples were scanned at wavelength of 0.1540 nm over a 3 to 80° angle range, using a 0.02-degree step size and a 0.4-s exposure per step.

#### Fourier-transform infrared spectroscopy

Fourier-transform infrared spectra (FT-IR) was recorded using KBr disc (4000–400 cm^−1^) on a Thermo-Nicolet IS10 FT-IR spectrometer (Nicolet Instrument Co, Madison, WI, USA) in transmission mode with a resolution of 4 cm^−1^.

#### Scanning *electron* microscopy/energy-dispersive X-ray analysis

SEM/EDX analysis was performed on a scanning electron microscope (Quanta 250FEG, FEI, USA).

#### Nitrogen adsorption isotherm

The BET surface area of the samples was obtained using a surface area and pore size distribution analyzer (BELSORP-miniX). The samples were then degassed at 120 °C for 24 h under nitrogen gas flow.

#### Magnetic saturation

Saturation magnetization of MBC was estimated using vibrating sample magnetometer (VSM, Lake Shore model 7410, USA), equipped with a 9 Tesla superconducting magnet (AboGabal et al. 2023).

#### Point of zero charge

The point of zero charge (pH_PZC_) was estimated using the pH drift approach. A series of bottles containing 20.0 mg of MBC and 50 mL of NaCl (0.1 mol L^−1^) solution, at initial pH (pH_initial_) ranged from 2.0 to 11.0, were agitated (150 rpm) at the room temperature (25.0 ± 1.0 °C) for 24 h. The shift in pH (ΔpH = pH_final_ – pH_initial_) was plotted against pH_initial_, and the pHpzc was estimated from the intersection of the *x*-axis.

### Batch adsorption experiments

Batch experiment was carried out to optimize the uptake process and to investigate the influence of various factors such as solution pH, sorbent dosage, contact time, and temperature on the adsorption performance. The experimental variables were chosen as follows: pH ranging from 2.0 to 11 using 0.5 mol L^−1^ solution of HCl or NaOH, sorbent dose ranging from 0.2 to 3.0 g L^−1^, contact time ranging from 5 to 120 min, and temperature ranging from 25 to 45 °C. The bottles were put in a temperature-controlled shaker at 150 rpm. After the adsorption process was completed, the MBC was collected at the internal side of the vessels by using an external magnet and the residual amount of MB was determined spectrophotometrically at 663 nm using a 7300 Genway UV–vis spectrophotometer. Finally, the removal percentage (*R*%) and equilibrium adsorption capacity (*q*_*e*_, mg g^−1^) were estimated using Eqs. 1 and 2, respectively.

1 $$R \left(\text{\%}\right)= \frac{{C}_{i}- {C}_{e}}{{C}_{i}} \times 100$$

2 $${q}_{e}= \frac{({C}_{i}- {C}_{e})V}{m}$$

where *C*_*i*_ and *C*_*e*_ are the concentrations of MB solution, in mg L^−1^, before and after adsorption, respectively; *V* (*L*) is the volume of the aqueous MB solution and m is the mass of the sorbent (*g*).

### Desorption study

The desorption of MB from MBC was evaluated by soaking 50 mg of MBC in 50 mL of 50 mg L^−1^ of MB solution. The mixture was shaken for 30 min to attain equilibrium state. After that, the sorbent was collected by the magnet and rinsed with distilled water to remove the excess dye. The collected sorbent was mixed with 10.0 mL of the under investigated eluent and shaken for 30 min. Finally, the sorbent was collected from the solution, rinsed again with distilled water, dried, and subjected to another cycle of adsorption/desorption to study the regeneration and reusability of MBC. The desorption percentage (*D*) can be estimated using Eq. (3):

3 $$D (\text{\%})=\frac{{C}_{d}{V}_{d}}{{(C}_{i}{ - C}_{e})V} \times 100$$

where *C*_*d*_ is the concentration of desorbed MB (mg L^−1^), *V*_*d*_ is volume of the eluent (*L*), and *V* is initial volume of the solution (*L*).

### Adsorption isotherm

The linear relationship that exists between the adsorption capacities and the equilibrium concentration of the adsorbate at homeostasis is represented by the adsorption isotherm. For this purpose, 20 mg of MBC was mixed with 50 mL of MB solution (concentration ranged from 5 to 200 mg L^−1^) and agitated as described above for 30 min. After that, the adsorbent was collected by a magnet and the remaining concentration of MB (*C*_*e*_) was determined spectrophotometrically. The Langmuir and Freundlich models were used to evaluate isotherm variables using Eqs. (4) and (5), respectively.

4 $$\frac{{C}_{e}}{{q}_{e}}=\frac{{C}_{e}}{{q}_{\text{max}}}+ \frac{1}{{q}_{\text{max}}{K}_{L}}$$

5 $${\text{ln}q}_{e}= {\text{ln}K}_{F }+ \frac{1}{{n}_{F}} {\text{ln}C}_{e}$$

where *C*_*e*_ and *q*_*e*_ are the equilibrium concentration (mg L^−1^) of the adsorbate and the equilibrium adsorption capacity (mg g^−1^), respectively, *q*_*m*_ is the maximum adsorption capacity of MBC towards MB in mg g^−1^, and *K*_*L*_ and *K*_*F*_ are the Langmuir constant and the Freundlich constant, both are expressed in L mg^−1^. 1/${n}_{F}$ is the heterogeneity factor and its value indicates the favorability of the adsorption process.

### Thermodynamic parameters

The enthalpy (Δ*H*) and entropy (Δ*S*) changes of adsorption were estimated using the van’t Hoff formula:

6 $$\text{ln}{K}_{d}= \frac{\Delta S}{R}- \frac{\Delta H}{\text{RT}}$$

where *K*_d_ (*q*_*e*_/*C*_*e*_) is the adsorption equilibrium constant (L g^−1^) and is estimated from the relation between *C*_*e*_ and *q*_*e*_ at different temperatures. *R* is the gas constant (8.314 J mol^−1^ K^−1^), and *T* is the absolute temperature (K). By plotting ln *K*_*d*_ versus 1/*T*, Δ*S* and Δ*H* can be determined from the intercept and the slope, respectively.

Additionally, the Gibbs free energy change (Δ*G*) for MB adsorption at temperatures of 25, 35, and 45 °C was computed using the following equation:

7 $$\Delta G=\Delta H-T\Delta S$$

## Results and discussion

### Characterization of biochar and magnetic biochar

#### XRD

**Fig. 1:**
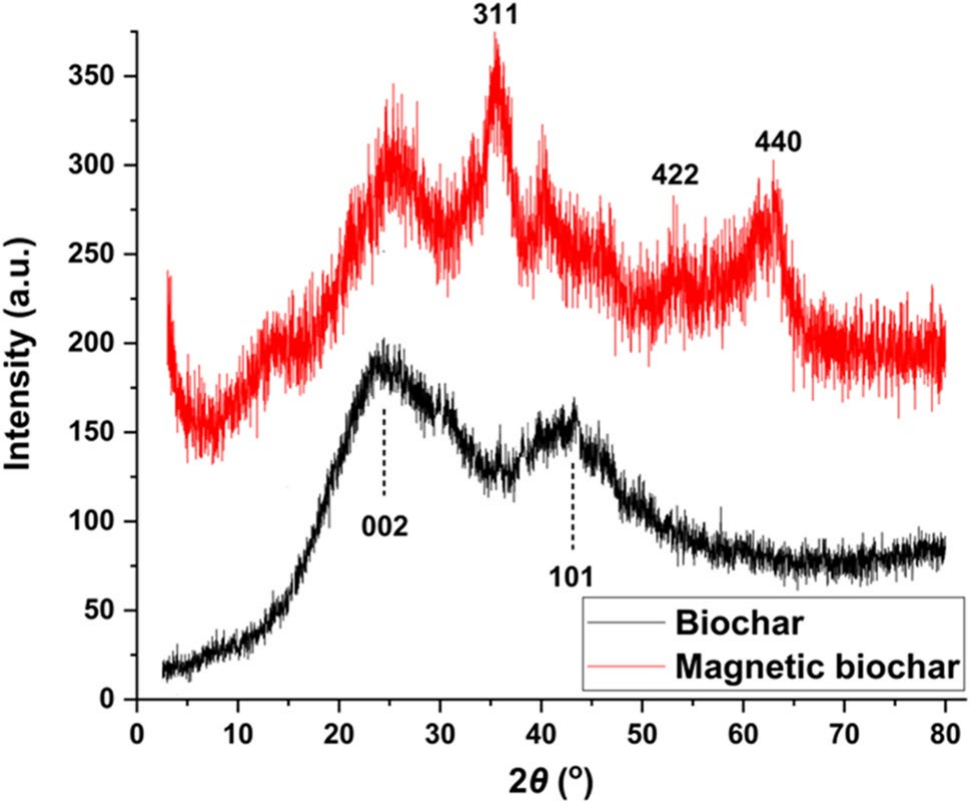
XRD patterns of biochar and magnetic biochar

Figure 1 presents the XRD patterns of the BC and MBC. As illustrated, the obtained biochar is amorphous, with broad diffraction peaks at 2*ϴ* = 24.5° and 43.2° attributed to the (002) and (101) plane of carbon, respectively (Altıntıg et al. 2017). New three characteristic peaks with 2*ϴ* values of 35.4° (220), 53.1° (422), and 63.0° (440) were observed after magnetization of the biochar, which were identified as Fe_3_O_4_ diffraction peaks according to the JCPDS Card No. 79–0417 (AboGabal et al. 2023). These findings confirmed the presence of Fe_3_O_4_ due to the magnetization of the biochar.

#### SEM/EDX analysis

**Fig. 2:**
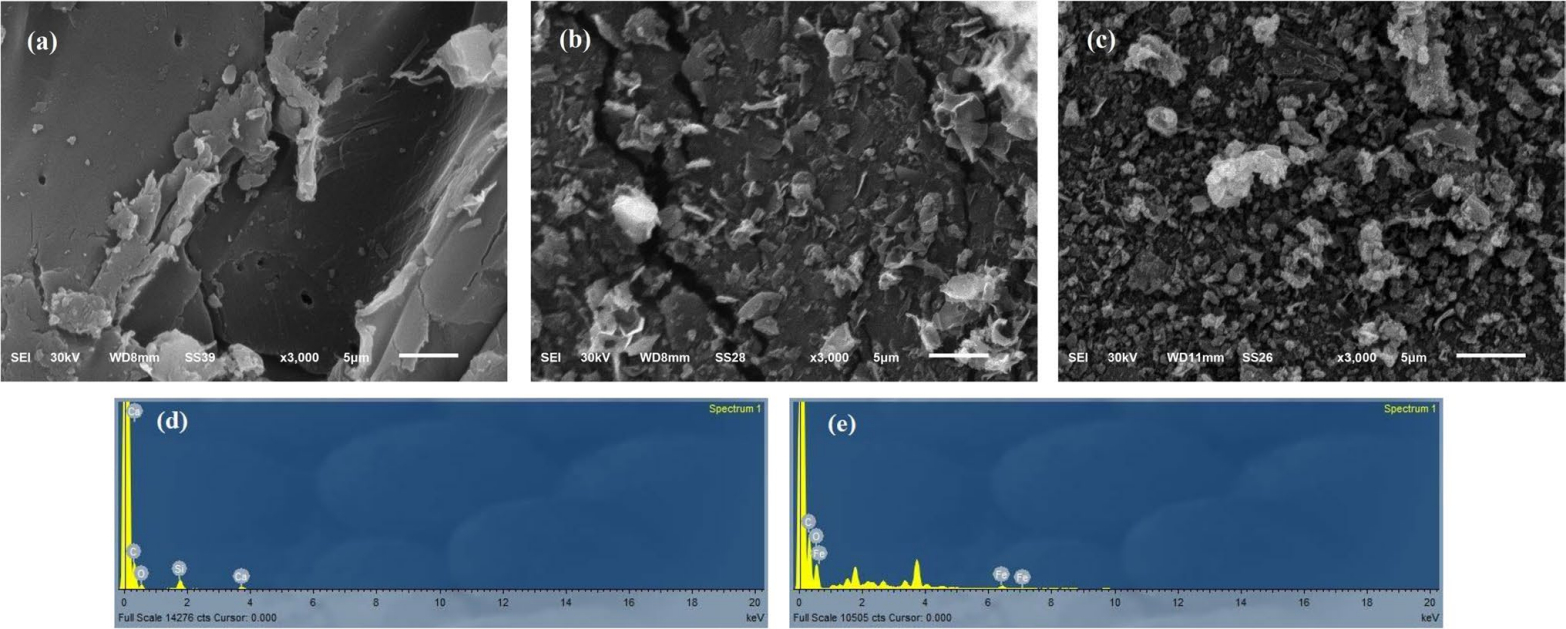
SEM analysis of **a** raw common reed, **b** BC, **c** MBC, and EDX analysis of **d** BC and **e** MBC

Figure 2 presents the SEM images of common reed, BC, and MBC. As is plainly seen in Fig. 2a and b, the surface morphology of common reed and BC differs significantly from one another. The surface of common reed has minimal or no cracking or porosity and is mostly smooth and flat. Upon calcination, a rough surface with deep cracks was obtained. The decomposition of lignin and lignocelluloses at elevated temperatures results in a development of pores (Liang et al. 2020). These cracks are evenly blocked by the numerous tiny particles on the surface (Fig. 2c), which are identified as Fe particles by the EDX analysis (Fig. 2e). The EDX analyses of BC and MBC are summarized in Table 1. Following the calcination process, the weight percentage of the oxygen element in the overall content dropped from 56.1% in the raw material % to 28.3%, in BC while the weight percentage of the carbon element increased from 43.9 to 65.7%. Additionally, the peaks of iron appeared in the spectrum of MBC (0.8%) suggesting successful incorporation of Fe_3_O_4_ in the structure of the biochar.

**Table 1:** EDX elemental analysis of raw common reed, BC, and MBC

Element	Common reed (wt%)	Biochar (wt%)	Magnetic biochar (wt%)
C	42.5	69.9	59.9
O	54.8	28.3	39.0
Fe	-	-	1.1
Si	1.4	1.3	-
Ca	0.5	0.5	-
K	0.8	-	-

#### FT-IR

**Fig. 3:**
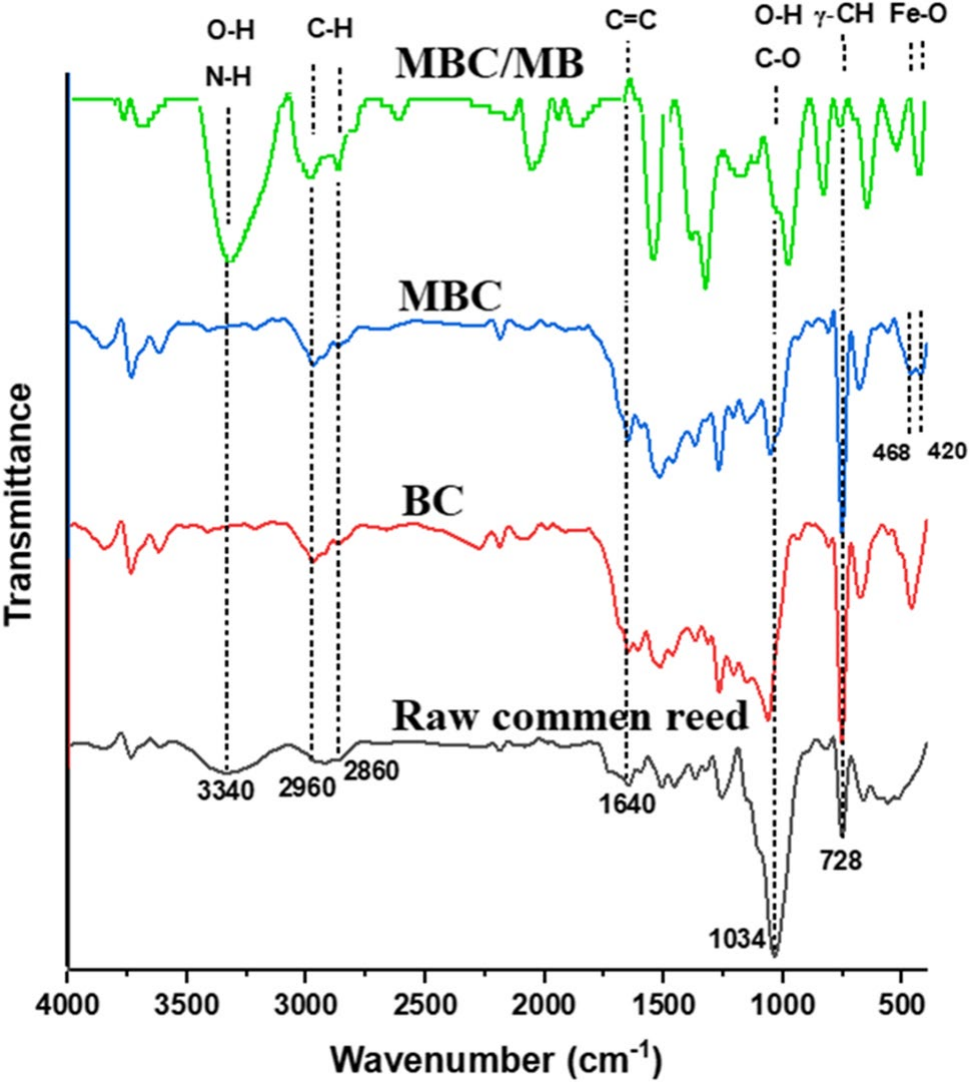
FT-IR of raw common reed, biochar, magnetic biochar, and magnetic biochar after adsorption of methylene blue

Figure 3 displays the FT-IR of common reed, BC, and MBC. The FT-IR chart of the common reed sample shows a band at 3340 cm^−1^ which is due to overlapping of the vibration stretching of O–H and N–H groups of the main components of the plant cells, i.e., lignin, cellulose, pectin, and hemicellulose (He et al. 2021). The weak bands at 2860–2960 cm^−1^ in the spectra of raw common reed, BC, and MBC were attributed to the aliphatic C–H stretch (Kang et al. 2019). The band at 1640 cm^−1^ was attributed to the stretching vibrations of aromatic C = C (Prakash et al. 2021). The band at 1034 cm^−1^ is associated with the O–H bending and C-O stretching of alcohols, phenols, carboxylic acids and esters (Hesas et al. 2013). The strong band at 748 cm^−1^ is due to γ-CH in the aromatic rings (Ge et al. 2023). After calcination, most of these peaks are kept or slightly shifted indicating that these active groups could reside on the surface of the biochar and provide chemical centers for uptake of MB. Moreover, two peaks related to the vibration of metal oxygen (Fe–O) bond emerged at 435 cm^−1^ and 535 cm^−1^ in MBC suggesting the presence of incorporated Fe_3_O_4_. Following the adsorption, the shifting of particular peaks from their pre-adsorption positions and/or changes in intensity suggested that the surface functional group had either weak van der Waals interactions or hydrogen bonding that contributed to the adsorption of MB molecules.

### BET analysis

**Fig. 4:**
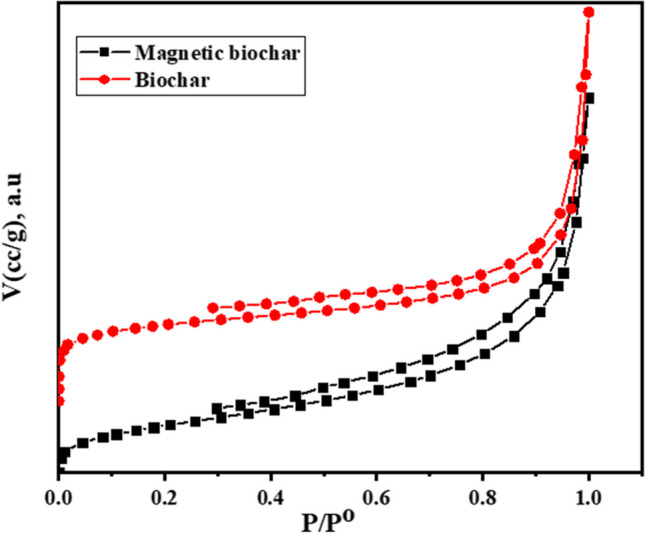
Nitrogen gas adsorption/desorption isotherm of BC and MBC at 77 K

BET study was utilized to estimate the specific surface area and pore size of both BC and MBC. As shown in Fig. 4, both BC and MBC exhibited a type II adsorption behavior based on the IUPAC classification with H3 hysteresis loops suggesting the presence of mesopores in the texture (Nouioua et al. 2023). The pore radii were 3.3 and 4.1 nm for BC and MBC, respectively, confirming the mesoporous structure of both materials (El Nemr et al. 2022). The specific surface areas of BC and MBC are 112.1 and 94.2 m^2^ g^−1^, respectively. The specific surface area of BC is slightly reduced after incorporation of Fe_3_O_4_ owing to blocking of pores and cracks. These findings are in good accord with the SEM analysis.

#### Saturation magnetism

**Fig. 5:**
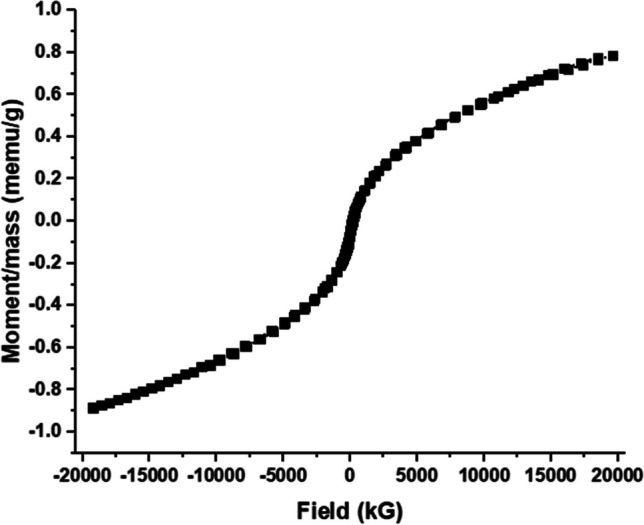
Magnetization curve of MBC

Magnetization curve was measured at 300 K to explore the magnetic separation characteristics of the prepared MBC (Fig. 5). The saturation magnetization of the sample was considerably low (at 0.79 emu g^−1^), due to the relatively low weight % of iron in the sample, i.e., 1.1%, based on EDX analysis. On the other hand, an external magnetic field potentially readily attracts the sorbent particles from the aqueous phase that can be removed by pipetting. These results are in good agreement with those of Zhu et al. who prepared magnetic porous carbon from hydrothermal biochar for removal of tetracycline (Zhu et al. 2014).

### Factors affecting adsorption

#### Initial pH and the point of zero charge

**Fig. 6:**
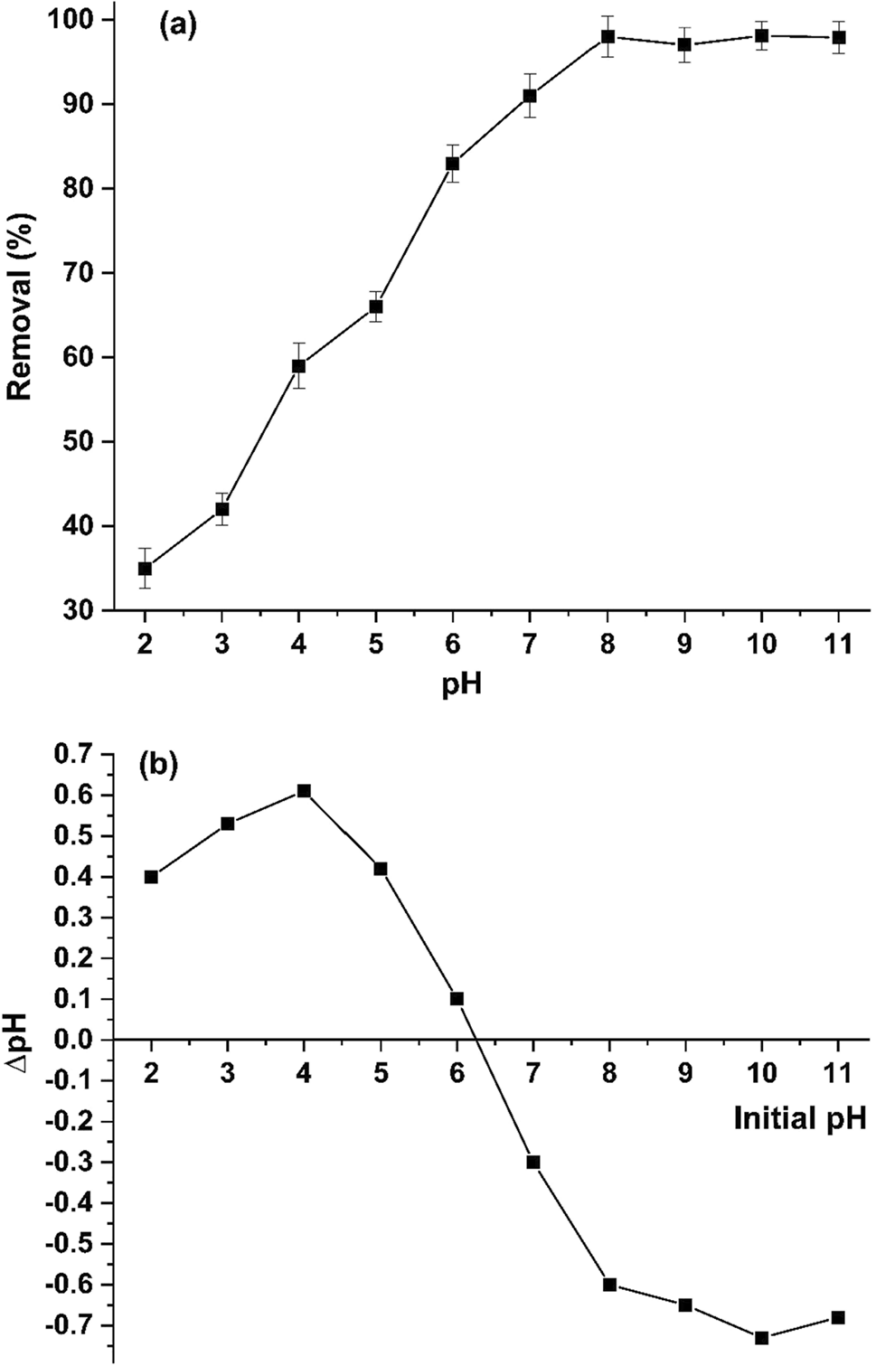
**A** Effect of pH on the adsorption of MB by MBC; **b** pH_PZC_ of MBC

The uptake of MB by MBC was investigated at wide pH range (2.0–11.0), and the results were displayed in Fig. 6a. The ionization of the adsorbate and the surface charge of the adsorbent are both influenced by the pH of the solution. At low pH, the uptake of MB is weak and after that it increased by increasing pH until reaching a maximum at pH 8.0. The results can be explained based on the pH_PZC_ which is the pH at which the net surface charge of the sorbent is neutral (Fiol and Villaescusa 2009). Therefore, its value is very important to estimate and explain the optimum pH for adsorption of MB by MBC. The value of pH_PZC_ of MBC was 6.2 as displayed in Fig. 6b. Below this value, hydronium ions (H_3_O^+^) produce a barrier that inhibits adsorption of MB onto MBC due to electrostatic repulsion. The protonated surface is not appropriate for the adsorption of the cationic dye. In contrast, at pH > 6.2, the surface of MBC acquires negative charges and therefore can adsorb MB via electrostatic attraction (Fito et al. 2023).

#### Adsorbent dose

**Fig. 7:**
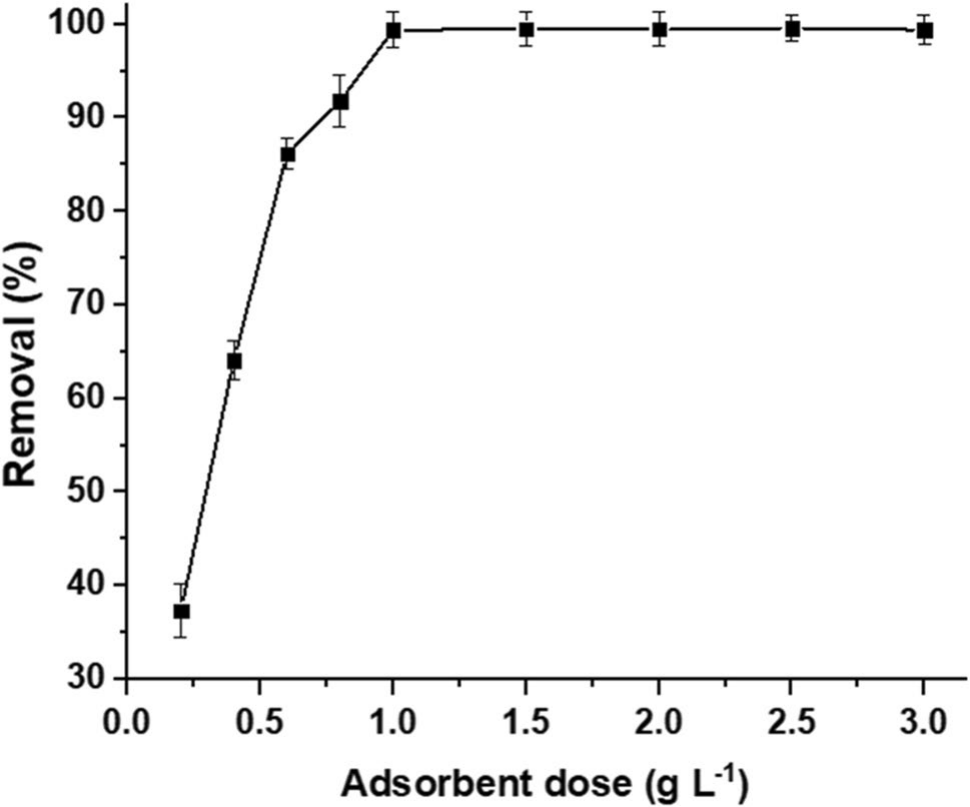
Effect of sorbent dose on the removal of MB by MBC

An important parameter that strongly affects the adsorbate-adsorbent equilibrium is the sorbent dose. The impact of MBC dose, from 0.2 to 3.0 g L^−1^, on the uptake of MB was displayed in Fig. 7. It was noted that when the sorbent dosage was raised from 0.2 to 1.0 g L^−1^, the removal rate rose considerably. After that, when the adsorbent amount was raised to 3.0 g L^−1^, the removal rate was maintained at almost 100%. The presence of active sites on the surface of MBC is the primary cause of this behavior (Ge et al. 2023). To attain maximum removal, the optimal sorbent dose of 1.0 g L^−1^ was determined.

#### Contact time

**Fig. 8:**
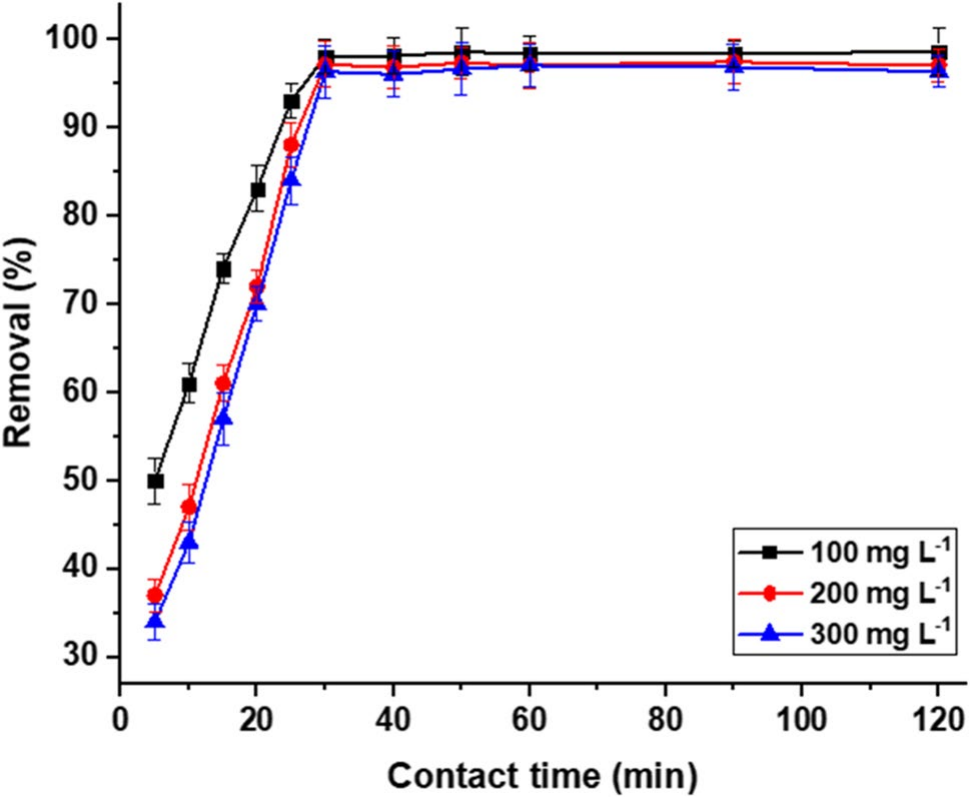
Effect of contact time on the removal of MB by MBC (pH 8.0, sorbent dosage: 1.0 g L.^−1^, contact time: (5 to 120 min) and room temperature: 25 ± 1 °C)

The consequence of contact time on the removal of MB by MBC was tested between 5 and 120 min, using a 50.0-mL solution containing different MB concentrations (100.0, 200.0, and 300.0 mg L^−1^), at pH 8.0, sorbent dose 1.0 g L^−1^, and at the ambient temperature. The obtained findings revealed that the uptake of MB dye increased rapidly at the beginning contact owing to the availability of active sites and reached equilibrium (removal = 96.0–99.0%) after 30 min (Fig. 8). The fast equilibrium is attributable to the electrostatic attraction between the active sites of MBC and MB. Adsorption of MB by MBC is quicker than previously produced bio-based sorbents such as 90 min for acid activated carbon derived from *Ficus carica* (Pathania et al. 2017), 180 min for pullulan polysaccharide/polyacrylamide/active carbon composite (Chen et al. 2022), and 360 min for nanocellulose bio-based composites (Oyarce et al. 2022).

#### Adsorption isotherms

**Fig. 9:**
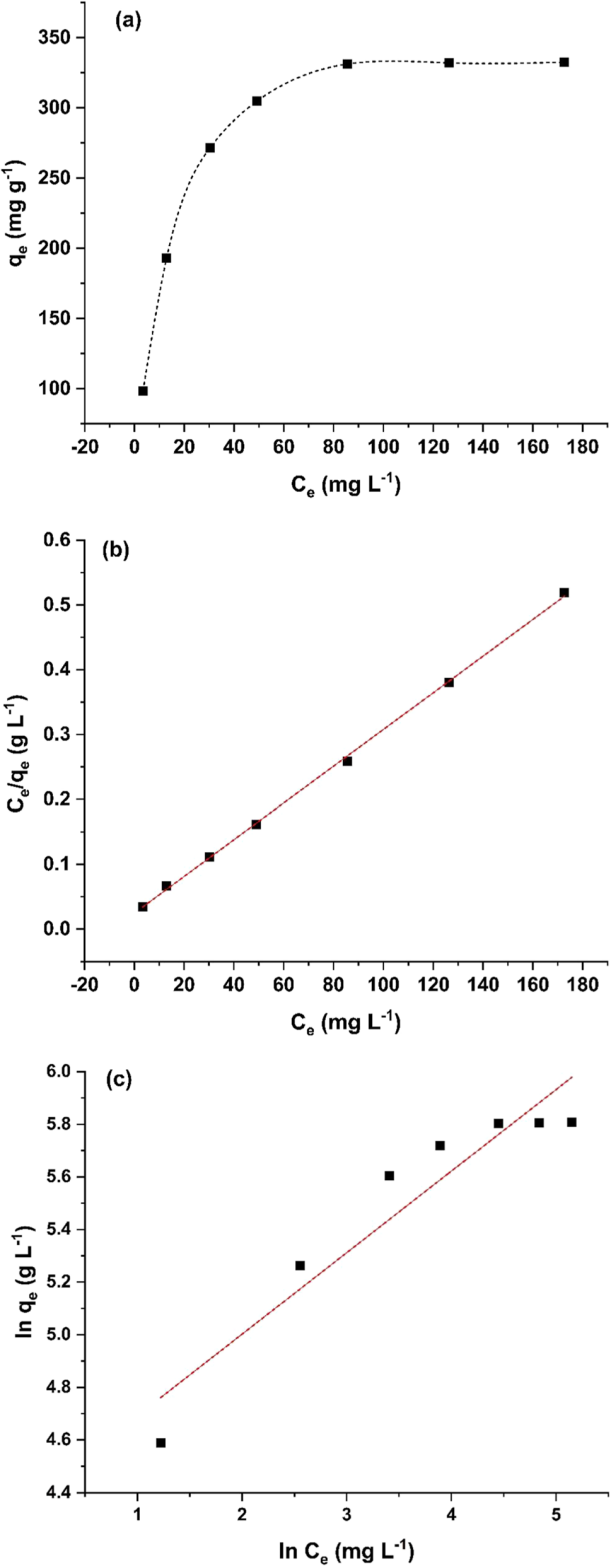
**A** Dependance of adsorption capacity of MBC on the equilibrium concentration of MB; **b** linear fitting curve of Langmuir model on the adsorption of MB by MBC; **c** linear fitting curve of Freundlich model on the adsorption of MB by MBC

The Langmuir and Freundlich isotherm equations are used to evaluate the data gained at equilibrium. Linear regression is commonly utilized to assess the practicality and best-fitting isotherm models. The Langmuir isotherm equation (Eq. 4) may be employed to estimate the maximum adsorption capacity (*q*_*m*_, mg g^−1^) of sorbent surface with full monolayer saturation, while the Freundlich model is applicable for heterogeneous surfaces and is described by Eq. 5 (El Nemr et al. 2021). Table 2 lists the fitting parameters, and Fig. 9 shows the estimated outcomes from the Langmuir and Freundlich models. As shown in Table 2, the correlation coefficient value (*R*^2^) of the Langmuir model was greater than that of the Freundlich model, indicating that the Langmuir model successfully matches the practical findings, and that the adsorption behavior was monolayer. The Langmuir equation predicted maximum adsorption capacity of MBC towards MB as 353.4 mg g^−1^ which was better than that of previously studied sorbents (Table 3).

**Table 2:** Fitting variables for Langmuir and Freundlich models

Langmuir				Freundlich		
*q*_*m*_ (mg g^−1^)	*K*_*L*_ (L mg^−1^)	*R*_*L*_	*R*^2^	*K*_*F*_ (L mg^−1^)	1/${n}_{F}$	*R*^2^
353.4	0.115	0.087	0.999	79.9	0.309	0.883

**Table 3:** Comparison of adsorption capacity of MB with other sorbents

Biosorbent	*q*_*m*_ (mg g^−1^)	Ref
KOH-activated porous biochar obtained from bamboo biochar	67.5	(Ge et al. 2023)
NaOH-modified mesoporous biochar produced from tea residue	105.3	(Mu and Ma 2021)
Porous biochar obtained by KOH activation of peanut shell biochar	208.0	(Han et al. 2015)
Activated carbon from pineapple biomass using ZnCl_2_ impregnation	288.3	(Mahamad et al. 2015)
Living biomass of the microalga *Chlamydomonas moewusii*	212.4	(Seoane et al. 2022)
Sulfuric acid-treated *white frangipani* leaf powder	250.0	(Deka et al. 2023)
Acid-factionalized biosorbent derived from coconut shell	50.6	(Jawad et al. 2020)
Cu-BTC metal organic framework	189.1	(Eren et al. 2020)
Zn-based metal organic framework	26.3	(Kumar et al. 2024)
Benzimidazole-based covalent organic framework	63.3	(Rahmanian et al. 2020)
Iron terephthalate metal–organic frameworks	187.0	(Haque et al. 2011)
Amine-functionalized metal organic frameworks	312.5	(Paiman et al. 2020)
Mesoporous magnetic biochar derived from common reed biomass	353.4	Our study

The nature of the adsorption process is dictated by factor *R*_*L*_, which is defined in Eq. 8.

8 $${R}_{L}=\frac{1}{{K}_{L}{C}_{i}}$$

The study shows that the value of *R*_*L*_ ranges from 0 to 1 and the *n*_*F*_ constant was more than the unity (Table 2), referring that the adsorption of MB onto MBC is a favorable phenomenon (Wang et al. 2023).

#### Thermodynamic indices

**Fig. 10:**
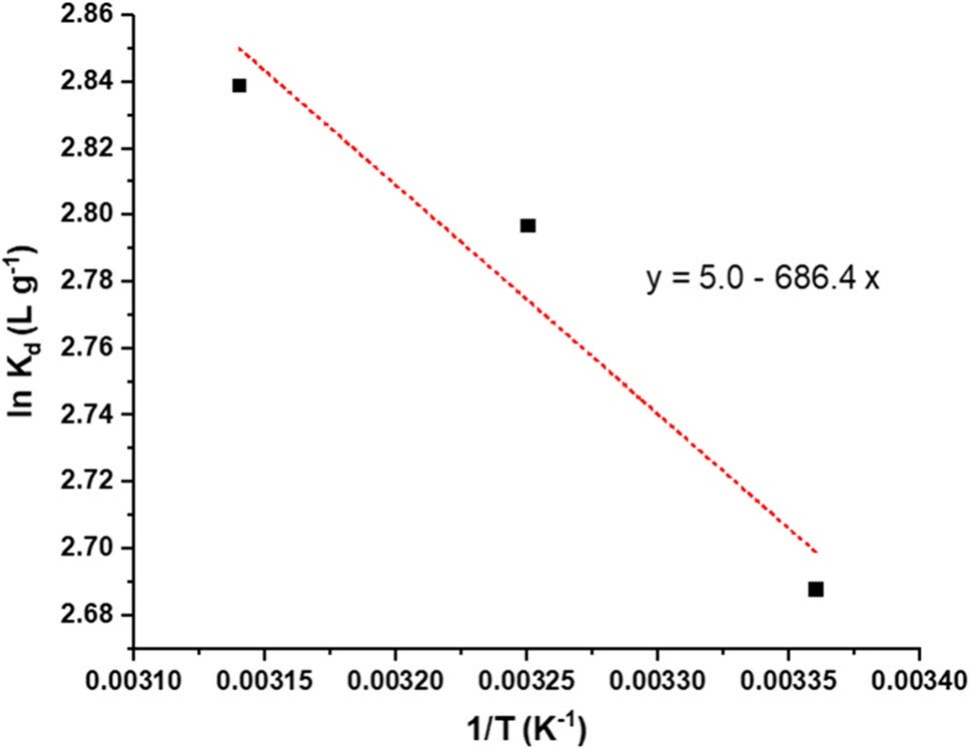
van’t Hoff plot of the adsorption of onto MBC.

For the adsorption of MB on MBC, several thermodynamic parameters were computed and summarized in Table 4. The negative signs of ΔG at all temperatures revealed that the adsorption was thermodynamically favorable and spontaneous. As well, the values of Δ*G* decreased with increasing temperature, showing that elevated temperatures can create a stronger adsorption driving force. The values of ∆*H* and ∆*S* estimated from van’t Hoff equation plot (Fig. 10) were 5.71 kJ mol^−1^ and 41.6 J mol^−1^ K^−1^, respectively. The positive sign of Δ*H* shows that the adsorption of MB onto MBC was endothermic, whereas the positive value of Δ*S* suggests an increase in randomness at the solid/solution boundary during the adsorption of MB onto MBC. When the Δ*H* is less than 25 kJ mol^−1^, the active force is typically van der Waals’ interaction, which is caused by physical adsorption; however, a value in the range of 40–200 kJ mol^−1^ suggested a chemical adsorption (Fan et al. 2017). In our work, the value of Δ*H* refers to prevalence of physical adsorption.

**Table 4:** Thermodynamic parameters of MB adsorption on MBC

Temperature (K)	298	308	318
∆*G* (kJ mol^−1^)	− 6.7	− 7.1	− 7.5
∆*H* (kJ mol^−1^)	5.71		
∆*S* (J mol^−1^ K^−1^)	41.6

#### Desorption and reusability

**Fig. 11:**
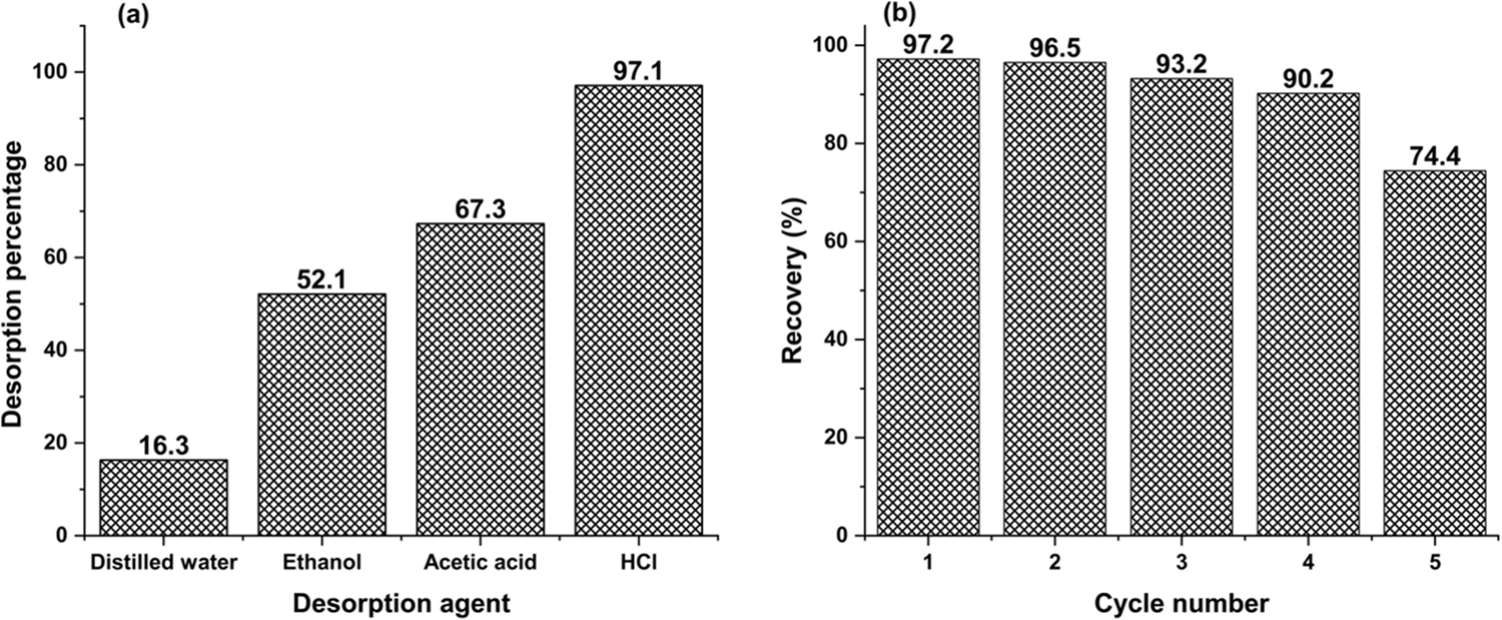
**A** Desorption and **b** reusability study (using 0.1 mol L^−1^ HCl) for adsorption of MB onto MBC.

The regeneration of the sorbent is a significant component in determining its application and economic values. Therefore, various solvents (distilled water, ethanol, acetic acid, and HCl) were employed to investigate the capacity to recover MB from the surface of MBC (Fig. 11a). As shown, the % desorption of HCl (0.1 mol L^−1^) > acetic acid > ethanol > distilled water. Therefore, 0.1 mol L^−1^ was selected as the optimum desorbing solvent. Furthermore, the adsorption efficiency of the regenerated MCB looked to recover mostly for the first four adsorption/desorption cycles but was somewhat reduced by about 25% after the fifth cycle (Fig. 11b). Therefore, the optimal recycling of MBC was four times.

### Proposed adsorption mechanism

The mechanism of MB removal by MBC is summarized in this section. In general, different types of interactions are responsible for the MB adsorption on the MBC surface. As shown in Fig. 6b, the pH_PZC_ of MBC is 6.2 indicating that the surface of the sorbent was negatively charged at pH values above 6.2. This enhanced the electrostatic attraction with the positively charged molecules such as MB. The mechanism of electrostatic adsorption aligns with the outcomes of previous investigations conducted on other materials (Abdulhameed et al. 2021; Ma et al. 2024; Zuhara et al. 2023). The FT-IR studies revealed the presence of hydroxyl, amide, carbonyl, and carboxyl groups in the structure of MBC that can act as H-bond donors or acceptors. The formation of H bonds between adsorbate and the active groups on the sorbent surface can cause either red or blue shifts in the FT-IR pattern (Mpatani et al. 2020). In this study, based on the changes observed in the FT-IR spectrum of MBC after adsorption of MB, it can be concluded that H-bond interaction is also a possible mechanism for the adsorption of MB onto MBC. The findings of thermodynamic analyses indicated that the adsorption process is spontaneous and endothermic. Moreover, the calculations of Δ*H* (5.71 kJ mol^−1^) suggested that the adsorption of MB onto MBC can also occur by van der Waal interactions.

## Conclusion

In this work, a low cost and effective adsorbent was prepared using common reed biomass as a source of carbon and then decorated with Fe_3_O_4_, resulting in a magnetic biochar. The prepared sorbent has good efficiency for adsorption of MB from aqueous medium. The adsorption isotherms revealed that the Langmuir model best suited the data, implying that MB adsorption occurred at a mono-layer process. The maximum adsorption capacity was 353.4 mg g^−1^ which was better than many biosorbents in the literature. Thermodynamic indicators suggested that MB adsorption by MBC was spontaneous and endothermic. The biosorbent can be reused up to four times without significant reduction in its adsorption efficiency. As a result, magnetic common reed-derived biochar is a potential and useful absorbent for removing MB from wastewater.

## Data Availability

The data that support the findings of this study are available from the corresponding author, upon reasonable request.
